# Patient positioning during pediatric cardiothoracic computed tomography using a high-resilience pad system and pre-scan measurement of chest thickness

**DOI:** 10.1038/s41598-022-21018-5

**Published:** 2022-10-05

**Authors:** Satoshi Higuchi, Tatsuya Nishii, Atsushi Hirota, Shota Harumoto, Hiroki Horinouchi, Emi Tateishi, Yasutoshi Ohta, Keisuke Kiso, Kenichi Kurosaki, Tetsuya Fukuda

**Affiliations:** 1 Department of Radiology, National Cerebral and Cardiovascular Center, 6-1 Kishibeshinmachi, Suita, Osaka Japan; 2 Department of Pediatric Cardiology, National Cerebral and Cardiovascular Center, 6-1 Kishibeshinmachi, Suita, Osaka Japan; 3 Department of Diagnostic Radiology, Tohoku University Hospital, 1 Seiryomachi, Aoba, Sendai, Miyagi Japan

**Keywords:** Cardiology, Medical research, Physics

## Abstract

Patient positioning at the isocenter of the CT gantry is important for optimizing image quality and radiation dose, but accurate positioning is challenging in pediatric patients. We evaluated whether the high-resilience pad and pre-scan measurement of chest thickness allow accurate positioning in pediatric patients with congenital heart disease. Sixty-seven patients aged 7 years or younger who underwent cardiothoracic CT were enrolled. The ideal table height, defined as the position at which the scanner’s and patient’s isocenters coincided, was determined by radiographers either manually (manual group) or based on the pad’s and chest’s thickness (calculated group). The distance between the two isocenters and image quality were evaluated. The calculated group demonstrated smaller isocenter distance and standard deviation (distance: 0.2 ± 5.8 mm vs. − 8.3 ± 11.6 mm, p < 0.01; absolute value: 4.1 [1.9–8.0] mm vs. 12.3 [5.1–16.3] mm, p < 0.01), and higher signal-to-noise ratio (SNR) and dose-normalized SNR (SNRD) in the descending aorta than the manual group (SNR: 39.8 [31.0–53.7] vs. 31.9 [28.9–36.6], p = 0.048, SNRD: 39.8 [31.0–53.7] vs. 31.9 [28.9–36.6], p = 0.04). The system allowed for more accurate positioning in pediatric cardiothoracic CT, yielding higher image quality.

## Introduction

CT has unparalleled advantages, including the rapid acquisition time, exquisite spatial resolution, and accurate three-dimensional evaluation of the complex CHD anatomy^1–3^. Nevertheless, using ionizing radiation, a high heart rate and body motion constitute well-documented drawbacks and limitations in pediatric patients^1,4^. Many techniques, such as high pitch scan, low tube voltage scanning, bowtie filtering, adaptive collimation, and iterative image reconstruction, have been introduced, aiming to optimize and reduce the radiation dose^5,6^. These advances in scanners have drastically reduced motion artifacts and the radiation dose, while the role of CT in managing CHD and planning interventions has been rapidly expanding^7–10^.

The main technique for avoiding image quality degradation and improper radiation exposure is the automated tube current modulation (ATCM), which optimizes the radiation dose by changing the tube current based on information obtained from projection localizer images^11–13^. For utilizing ATCM properly, patient positioning at the isocenter of the CT gantry is important^1,5,6^. If the patient is positioned off-center, the radiation dose estimated by the ATCM increases or decreases resulting in image quality degradation or higher radiation dose^12,14–17^.

The vertical off-centering by 20 mm on chest CT was reported to result in 7% organ dose differences, while off-centering by > 40 mm is associated with significant dose differences of ≥ 20%^18^. Although radiographers can visually check the patient’s central positioning using laser beams on the scanners, accurate positioning is difficult. The past studies demonstrated that inappropriate patient positioning of > 10 mm from the isocenter of gantry is observed in over 75% of patients and the mean distance from the isocenter is 33 mm^19,20^. In pediatric patients, accurate positioning is more challenging due to the small body size and the vacuum device wrapped around their body, used to immobilize patients, to reduce motion artifacts and the depth of sedation. Moreover, for the examination of small pediatric patients, additional pads or cushions are required to lift the patients to the isocenter because of the limited table height in the CT scanner. We previously placed towels or soft cushions, but the patient’s position within the scan lengths fluctuated.

We hypothesized that the patient’s position can be controlled by utilizing a high-resilience pad that prevents sinking of the body into the mat. Thus, we aimed to evaluate whether such a pad system and a pre-scan measurement of chest thickness could improve the patients’ positioning during cardiothoracic CTA and the signal-to-noise ratio (SNR) in pediatric patients with CHD.

## Methods

### Patients

This retrospective study was conducted in accordance with the guidelines of the Declaration of Helsinki (Washington, World Medical Association, 2013) and approved by the research ethics committee of National Cerebral and Cardiovascular Center (approval #R19039-2). We did not obtain written informed consent from the patients according to Ethical Guidelines for Medical and Health Research Involving Human Subjects^21^ and the research ethics committee of National Cerebral and Cardiovascular Center waived the requirement of informed consent. Seventy consecutive pediatric patients with CHD, aged 7 years or younger, who underwent cardiothoracic CT using a third-generation dual-source CT scanner (SOMATOM Force, Siemens Heathineers, Forchleim, Germany) between October 2019 and May 2020 were included. Patients in whom the cervical or abdominal region was imaged in addition to the thoracic region were excluded. Patient demographic characteristics, including sex, age, body weight, height, and pulse rate (measured at CT acquisition), were collected. Patients were divided into two groups: the first half of the patients were assigned to the “manual” group and the second half in the “calculated” group.

### Patient positioning

**Figure 1:**
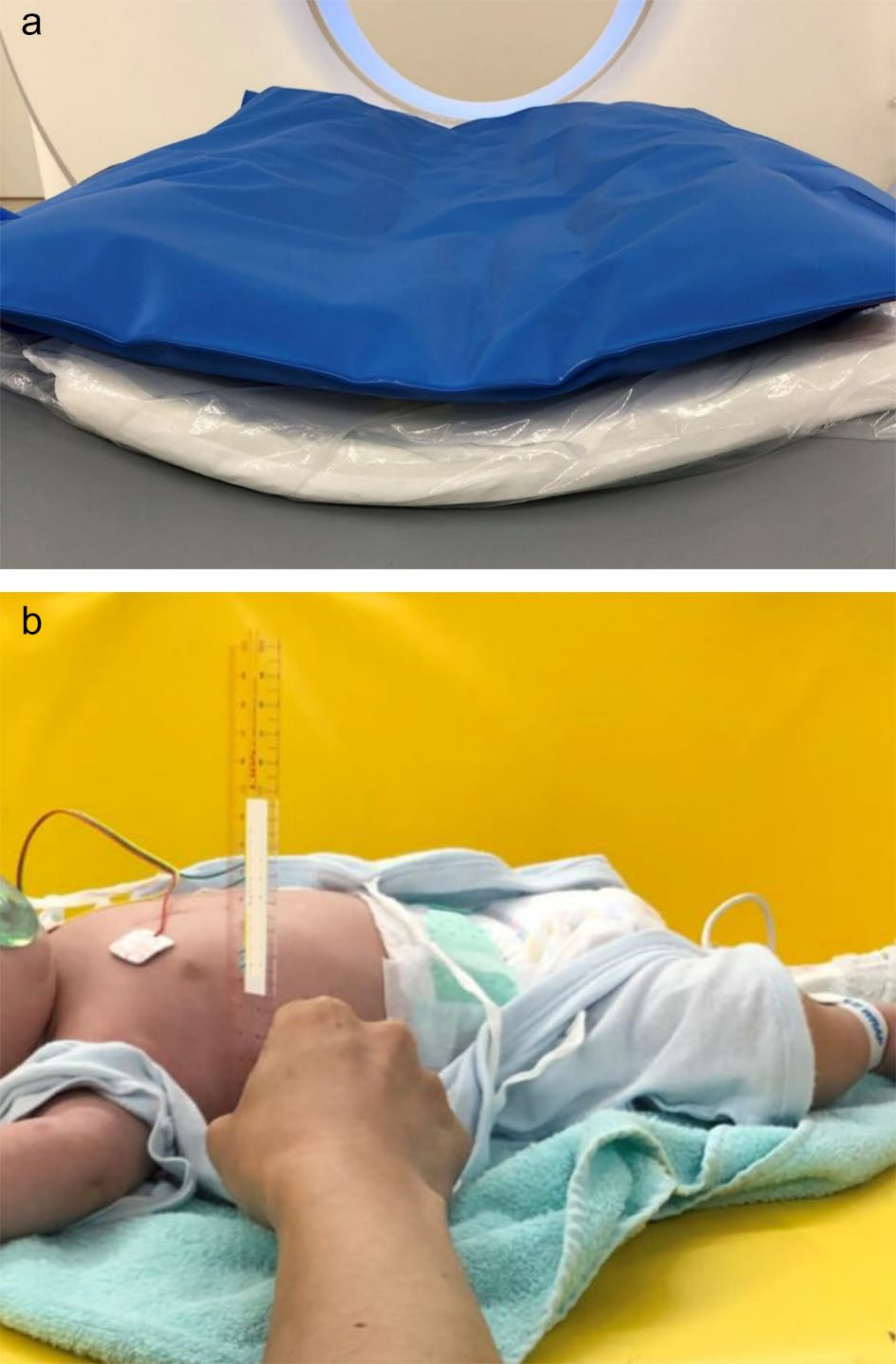
(**a**) The high-resilience pad (white mattress) is put under the vacuum device (blue mat). The total thickness of the pad system was measured at 75 mm from the top plate, which is restricted to move up to 100 mm below the isocenter of the gantry. (**b**) The chest thickness in the supine position was measured at the level of the nipple by a ruler. In the calculated group, the setting table height was calculated using the following formula; setting table height = 75 + measured chest thickness (mm)/2.

We applied a high-resilience pad in addition to a vacuum device (MedVac Vacuum Immobilization Splints, KOHLBRAT&BUNZ GKBH, Radstadt, Austria) on the CT table for all patients (Fig. 1a). We scanned the pad system before the study and measured its total thickness as 75 mm. Chest thickness was defined as the vertical distance at the level of the nipple and measured by a ruler in the supine position (Fig. 1b). Since the table height of the scanner indicates the distance (mm) from the isocenter to the tabletop, in the calculated group, the ideal table height was calculated based on the measured chest thickness and thickness of the pad system as follows: setting table height = 75 + chest thickness (mm)/2 (Supplemental Figure). In the manual group, radiographers manually adjusted the table height using laser beams on the scanner.

### CT scan protocol

Image acquisition included three consecutive scans: anterior-to-posterior (AP) scout scan, monitoring scan, and cardiothoracic CT angiography (CTA). All patients received an intravenous injection of 740 mgI/kg of non-iodinated contrast agent (iopamidol, Iopamiron 370 mgI/mL; Bayer, Osaka, Japan), followed by a saline chaser, using a contrast injector (DUAL SHOT GX 7, Nemoto Kyorindo, Tokyo, Japan). In patients under 10 kg, the contrast agent was diluted to 50%. The injection time of the contrast agent and the scan delay time from the start of the injection were changed according to the hemodynamics: 25 and 45 s for Glenn shunt, 30 and 75 s for Fontan circulation, and 18 and 25 s for others, respectively.

The cardiothoracic CTA with non-electrocardiogram-triggered, high-pitch (pitch value: 3.2) spiral acquisition was performed using the following parameters: reference tube voltage, 80 kV; reference effective tube load, 240 mAs, with automated tube voltage selection (CARE kV, Siemens); collimation, 192 × 0.6 mm; gantry rotation time, 250 ms. The CT images obtained by advanced modeled iterative reconstruction (ADMIRE) were reconstructed using a display field of view of 160–240 mm according to patient physique, slice thicknesses of 5 and 1 mm, and a vascular standard reconstruction kernel of Bv40. The noise reduction level of ADMIRE was set to 3 and 4 for 5-mm and 1-mm slices, respectively. For image analysis, another set of images were reconstructed using a maximum display field of view of 354 mm so that the center of the image coincided with the isocenter of the gantry.

### Image analysis

**Figure 2:**
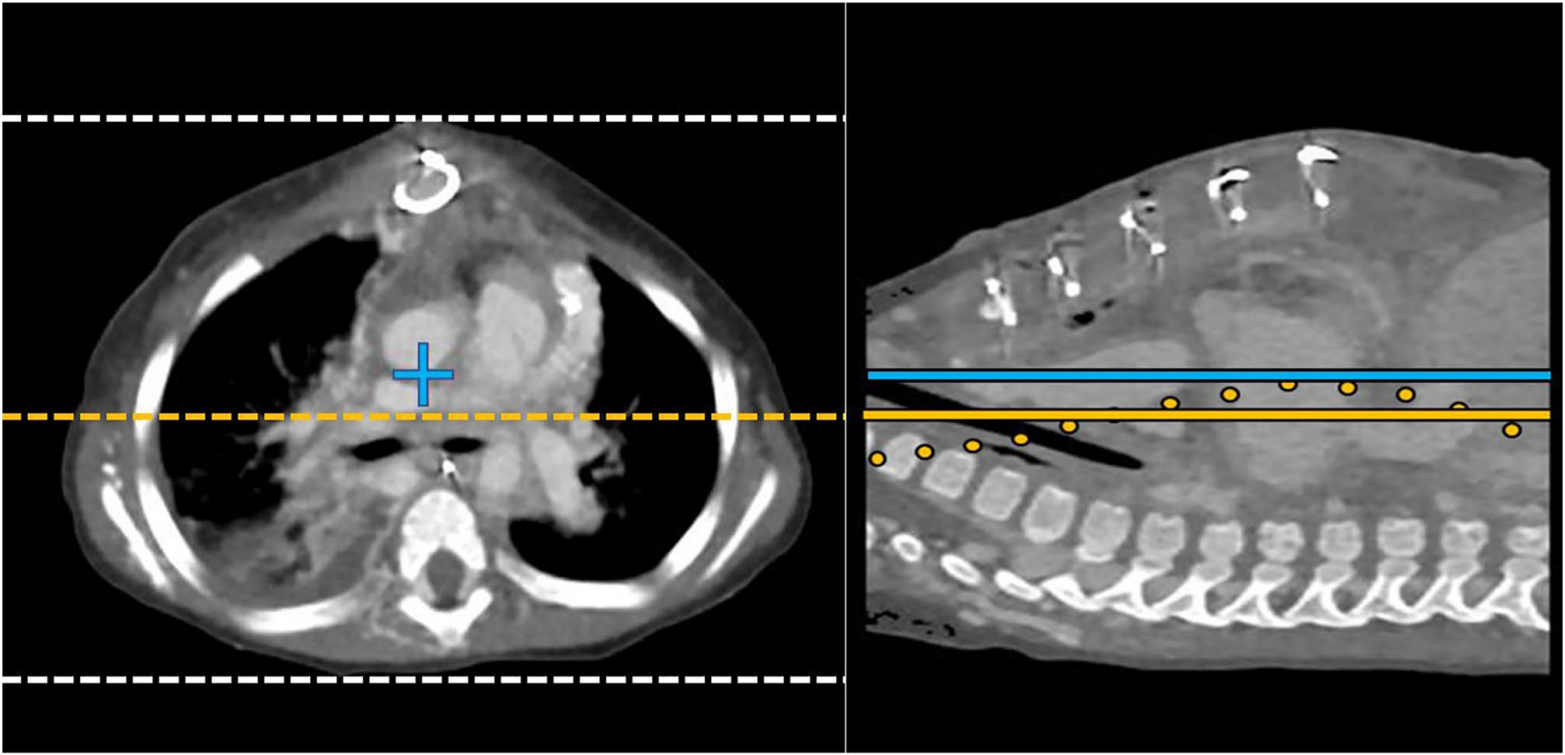
Definition of patient isocenter. (**a**) Axial image binarized with the CT value threshold to extract the patient isocenter, defined as the middle point (yellow dotted line) between the highest and lowest point (white dotted lines) of the extracted skin surface. All images were reconstructed so that the center of the image coincides with the isocenter of the gantry (blue cross). (**b**) Sagittal image showing the grand truth vertical center of patients, calculated using the averaged center values of each image along the scan length (yellow line). The patient was positioned below the isocenter of the gantry (blue line).

The following image processing and analysis were performed by a radiologist with 7 years of experience. First, binarization of CT images was performed: the skin contour was extracted by thresholding segmentation using a workstation (Ziostation2; Ziosoft, Tokyo, Japan). The vertical center of the patients in each slice was defined as the middle position between the highest and lowest points of the extracted skin surface. The grand truth vertical center was calculated as the averaged center values of each image along the scan length (Fig. 2). The distance between the isocenter of the CT gantry and the ground truth patients’ center (isocenter distance) was calculated based on the coordinate distance and matrix size (354/512 mm per pixel) using an in-house calculator program written in Python-3.7.5, NumPy-1.17.3 (available at http://www.python.org/, http://www.numpy.org/). The isocenter distance was indicated with a sign; positive and negative values mean positioning above and below the isocenter, respectively. Body thickness and width were defined as the averaged thickness along the scan length and the distance between right and left ends in the middle slice, respectively, to calculate size-specific dose estimates (SSDE)^22^. We also assessed the isocenter distance to body thickness ratio (distance ratio) and the number of patients with an offset > 20 mm in each group. Subjective image quality was evaluated by a cardiovascular radiologist (T.N.) with 14 years of experience in pediatric and cardiovascular radiology for all patients. Overall image quality was evaluated on the following 4-point scale: 4 = good quality with low noise, 3 = fair quality with moderate noise but suitable for diagnosis, 2 = poor quality with strong noise and not suitable for diagnosis, and 1 = bad quality with strong noise and inability to separate cardiovascular structures. The signal-to-noise ratio (SNR) in the ascending and descending aorta was evaluated in an image with slice thickness of 1 mm at the level of the tracheal bifurcation in patients with native circulation. Dose-normalized SNR (SNRD) was also evaluated to reduce the effect on SNR by ATCM exposure reduction caused by off-center positioning. SNR and SNRD were calculated as the mean CT value/mean SD in regions of interest and SNR/√Dose, respectively.

For estimation of the fluctuation of the pad system, its average thickness was calculated based on the table height and above parameters.

### Statistical analysis

Descriptive statistics are presented as mean ± standard deviation (SD) for continuous variables and as numbers for categorical variables. Non-normally distributed variables are shown as medians with interquartile ranges (IQR). The normality of CT parameters and demographic data was tested using the Shapiro–Wilk test (*p* > 0.05). Patient demographic data, radiation dose, accuracy of patient positioning, and image quality were compared between groups using the Wilcoxon signed-rank test. For analyzing between-group differences in the proportions of patients with isocenter offset > 20 mm, the χ^2^ test was applied. The Pearson correlation coefficient was used to evaluate the correlations between the measured chest thickness and body thickness derived from CT images.

Statistical analyses were performed using JMP Pro version 14.2.0 (SAS Institute Inc., Cary, USA). P*-*values < 0.05 were considered statistically significant.

## Results

### Patient characteristics

Seventy patients who underwent cardiothoracic CT were divided into the manual and the calculated group. Three patients were excluded as the scan included the cervical (n = 1) or abdominal (n = 2) region in addition to the thoracic region. Thus, 34 patients in the manual group and 33 in the calculated group were included in the analysis. The manual group included 23 patients with native circulation, 4 with Glenn shunt, 3 with Fontan circulation, and 4 with postoperative infection. The respective numbers in the calculated group were 21, 6, 4, and 2. The baseline patient characteristics are summarized in Table 1. Demographic data showed no significant differences between groups. No significant differences were found in the physical parameters obtained from CT images, including body thickness, width, and effective diameter, as well as radiation dose parameters, such as the CT dose index volume (32 cm), dose-length product, and SSDE.

**Table 1:** Patient demographics.

	Manual group (n = 34)	Calculated group (n = 33)	P value
Age (months)	8 [2–31]	10 [1–29]	0.99
Female	17	19	0.63
Body weight (kg)	6.4 [3.3–8.8]	6.5 [3.3–10.5]	0.77
Height (cm)	64 [51–86]	65 [52–85]	0.67
Body thickness (mm)	104 [96–119]	108 [94–122]	0.76
Body width (mm)	152 [113–173]	154 [116–184]	0.56
Effective diameter (cm)	12.2 [10.3–14.4]	13.0 [10.3–15.0]	0.58
Heart rate (bpm)	120 [109–143]	134 [121–149]	0.07
CTDIvol(16 cm) (mGy)	0.72 [0.54–1.06]	0.7 [0.52–1.08]	0.6
DLP (mGy･cm)	6.0 [4.2–10.5]	5.9 [3.7–10.0]	0.36
SSDE (mGy)	0.76 [0.64–1.16]	0.90 [0.64–1.38]	0.32

Values are n, median [interquartile range]. * indicates statistical significance.

*CTDIvol* computed tomography dose index volume, *DLP* dose-length product, *SSDE* size-specific dose estimates (14).

### Patient positioning accuracy

**Figure 3:**
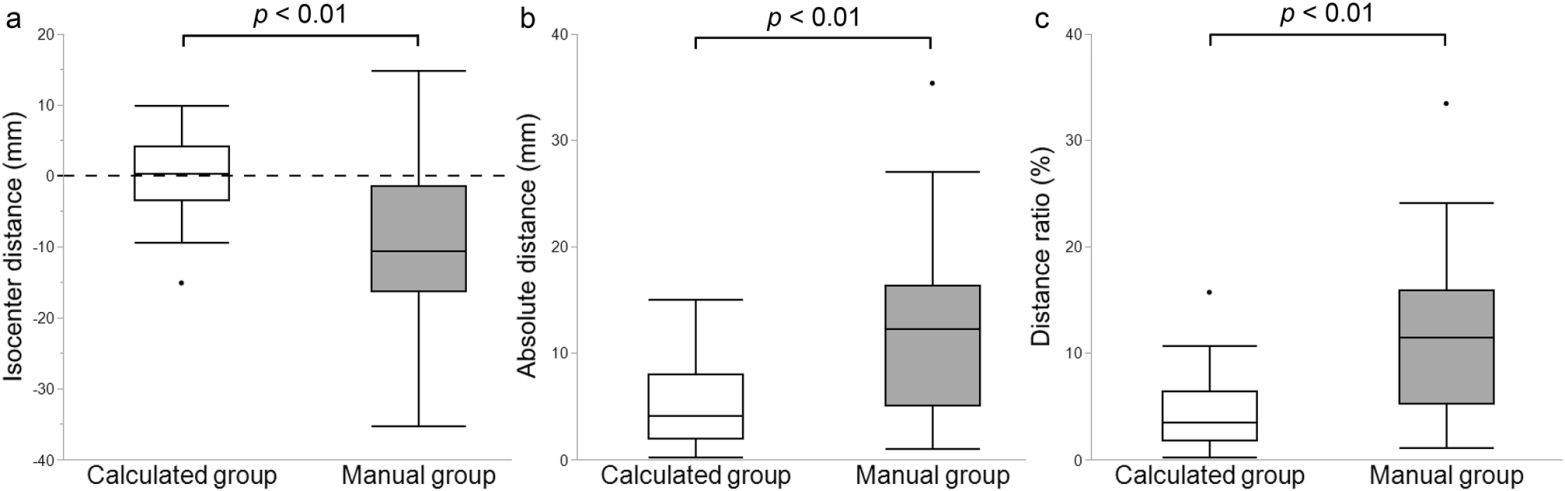
Box-and-whisker plots of patient positioning performance in the calculated and manual groups. The median (horizontal line within box), interquartile range (box), and nonoutlier range (whiskers) defined as 1.5 times the interquartile range. The largest deviations from the scanner isocenter, outside the nonoutlier range, are plotted as open dots. (**a**) Isocenter distance. The positive and negative values above and below the isocenter, respectively. (**b**) Absolute distance. (**c**) Distance ratio, defined as the ratio of the absolute isocenter distance to the body thickness.

The isocenter distance and SD were significantly smaller in the calculated than in the manual group (0.2 ± 5.8 mm vs. − 8.3 ± 11.6 mm, p < 0.01) (Fig. 3a). The absolute value of isocenter distance was 4.1 [1.9–8.0] mm and 12.3 [5.1–16.3] mm in the calculated and manual group, respectively (p < 0.01) (Fig. 3b). The distance ratio (0.2 ± 5.3% vs. 7.8 ± 11.3%, p < 0.01) was also smaller in the calculated group (Fig. 3c). The isocenter offset was > 20 mm in four patients of the manual group but none of the calculated group (p = 0.04).

### Image quality

The subjective image quality was rated as good, fair, and poor for 19 (58%), 14 (42%), and 0 (0%) patients in the calculated group, and 18 (53%), 15 (44%), 1 (3%) in the manual group, resulting in no significant difference between the two groups. In addition, 23 patients in the manual group and 21 in the calculated group who underwent cardiothoracic CTA with native circulation were included in the quantitative image quality analysis. The SNR and SNRD in the descending aorta were significantly higher in the calculated than in the manual group (SNR: 39.8 [31.0–53.7] vs. 31.9 [28.9–36.6], p = 0.048; SNRD: 39.8 [31.0–53.7] vs. 31.9 [28.9–36.6], p = 0.04), whereas no significant differences in the SNR or SNRD were found in the ascending aorta (SNR: 35.6 [26.2–56.4] vs. 34.4 [26.3–55.3], p = 0.52; SNRD: 64.1 [46.7–94.2] vs. 56.2 [40.8–78.7], p = 0.29) (Table 2).

**Table 2:** Image quality in the aorta.

	Calculated group (n = 21)	Manual group (n = 23)	P value
**Ascending aorta**			
SNR	35.6 [26.2–56.4]	34.4 [26.3–55.3]	0.52
SNRD	64.1 [46.7–94.2]	56.2 [40.8–78.7]	0.29
**Descending aorta**			
SNR	39.8 [31.0–53.7]	31.6 [28.9–36.6]	0.048*
SNRD	81.1 [49.0–106.4]	59.1 [47.5–68.0]	0.04*

The patients with Glenn shunt and Fontan circulation are excluded due to their specific methods of contrast agent administration. * indicates statistical significance.

*SNR* signal-to-noise ratio, *SNRD* dose-normalized signal-to-noise ratio.

### Difference between measured chest thickness and body thickness derived from CT images

In the calculated group, the average values of measured chest thickness and body thickness derived from CT images were 108.7 ± 20 mm and 101.5 ± 15.9 mm, respectively, with an average difference of 7.2 mm [95% confidence interval (CI): 3.7–10.7 mm] and a good positive correlation (R^2^ = 0.76, p < 0.01).

### Fluctuation of the pad system thickness

The average thickness of the pad system, as derived from CT images, was 71.6 ± 3.9 mm. The original thickness of the pad system was measured at 75 mm based on CT images acquired before this study, indicating an average fluctuation of 3.4 mm (95% CI: 2.0–4.8 mm).

## Discussion

In this study, the patients’ positioning during cardiothoracic CT was significantly more accurate when using the high-resilience pad system and pre-scan measurement of chest thickness than when manual adjusting the position in our pediatric patients with CHD. Furthermore, the position of patients was below the isocenter of the gantry and the SNR and SNRD in the descending aorta were lower in the manual than in the calculated group.

To our knowledge, no previous studies have evaluated whether accurate table height can be predicted from pre-scan measurement of chest thickness using the high-resilience pad system for the positioning of small pediatric patients. In adult thoracic CT, the absolute offset value of manual positioning was reported at 15–35 mm. Previous studies have also shown a higher probability of off-centering in slim or small patients than in obese or large patients^14,20^. In the examination of small pediatric patients, immobilization devices result in poorer visibility of the patient’s body outline and make accurate positioning difficult. In this study, the manual positioning demonstrated an offset of 12.3 [5.1–16.3] mm, indicating that radiographers achieved a smaller offset than did those in a previous study^14^, however, the manual group showed lower SNR and SNRD in the descending aorta. The reason for it can be attributed to the effect of both ATCM and bowtie filters in the off-center positioning. If the patient is located below the isocenter, the patient size estimated from the scout scan image will be smaller than the actual size, resulting in reduction in radiation dose and image quality by ATCM, but there was no statistical difference in the dose and SNR in the ascending aorta between the two groups. The bowtie filter reduces x-ray intensity especially toward the periphery of the image by spatially shaping the x-ray field intensity within the scan field of view independent of dose reduction with ATCM^17^. Therefore, although there was no statistical difference in the overall radiation dose, the bowtie filter possibly induces the excessive radiation dose reduction in the descending aorta which was located farthest from the isocenter in the manual group, resulting in a lower SNR. The higher SNR of the descending than ascending aorta in the calculated group might be due to higher SD in the ascending aorta by streak artifacts from the high concentration of contrast agent in the SVC and motion artifacts from heartbeats. Johnson et al. demonstrated that the overall radiation exposure in children with heart diseases is relatively low (median cumulative effective dose, 2.7 mSv), whereas cardiac catheterization and CT account for 81% of the cumulative exposure^4^. We believe that this method could be an option based on the "Image Gently" concept, as it is a simple method that allows isocenter positioning with minimal effort and without additional radiation exposure, as well as improved quantitative image quality.

The results showed 7-mm error in the measured body thickness (overestimate) and a 3-mm fluctuation in the pad system (dent), which resulted in a SD of 5.8 mm for the offset in the calculated group. Although other available methods for accurate measurement of body thickness can improve the error and SD, our system achieved an isocenter offset < 20 mm for every patient and a smaller SD than did manual positioning. Radiographers usually have the duty to perform the procedures in a relatively short time and thus at the expense of quality of clinical examinations. In this system, the ideal table height can be calculated from the equation using the chest thickness measured before the CT examination, thus reducing the examination time and the caveats of manual positioning.

The addition of a lateral scout scan can also improve patient positioning^14^; however, the scan requires additional radiation exposure for patients and examination time. Past studies have shown that a 3D camera for body contour detection allows for more accurate adult patient centering as compared with that achieved by manual positioning^23,24^. Recently, Booij et al. reported similar findings in pediatric patients^25^. The authors trained the algorithm using a large dataset of pediatric patients to improve the detection of small body contour and adjust the body model for these patients, since such an algorithm is not installed in the commercially available 3D camera. They found that pediatric patient positioning was significantly more accurate when using the 3D camera than when using manual adjustments in patients without baby cradle or vacuum cushion. In contrast, the positioning of patients with baby cradle or vacuum cushion was not improved by their system: the median (IQR) absolute table height deviation in thorax CT scans was 15.2 (15.0) mm and 15.3 (15.8) mm in the 3D camera and manual positioning group, respectively. In this study, the vacuum device was applied to all subjects, indicating that our method can offer more accurate positioning for patients who are challenging for 3D cameras and radiographers. Moreover, the advantage of our method is its high availability and feasibility for every institution where pediatric CT is performed routinely.

This study had several limitations. First, it was a retrospective, observational, single-center study, with a risk of selection bias. Second, the sample size was relatively small. A larger study is warranted to confirm our results.

## Conclusion

We demonstrated that, compared to manual patient positioning, the high-resilience pad system and pre-scan measurement of chest thickness allow radiographers to more accurately position small pediatric patients who require the use of a vacuum device at the isocenter of the CT gantry during cardiothoracic CT. Further this system allows the assessment of CHD, resulting in a higher SNR and SNRD in the descending aorta.

## Supplementary Information

Supplementary Figure S1.

Supplementary Legends.

## Data Availability

The data that support the findings of this study are available from the corresponding author, S.H., upon reasonable request.
