# Supplementary figures and images for: Patient positioning during pediatric cardiothoracic computed tomography using a high-resilience pad system and pre-scan measurement of chest thickness
Source: Sci Rep. 2022 Oct 5;12:16618. doi: 10.1038/s41598-022-21018-5 (PMC9534888; doi:10.1038/s41598-022-21018-5)

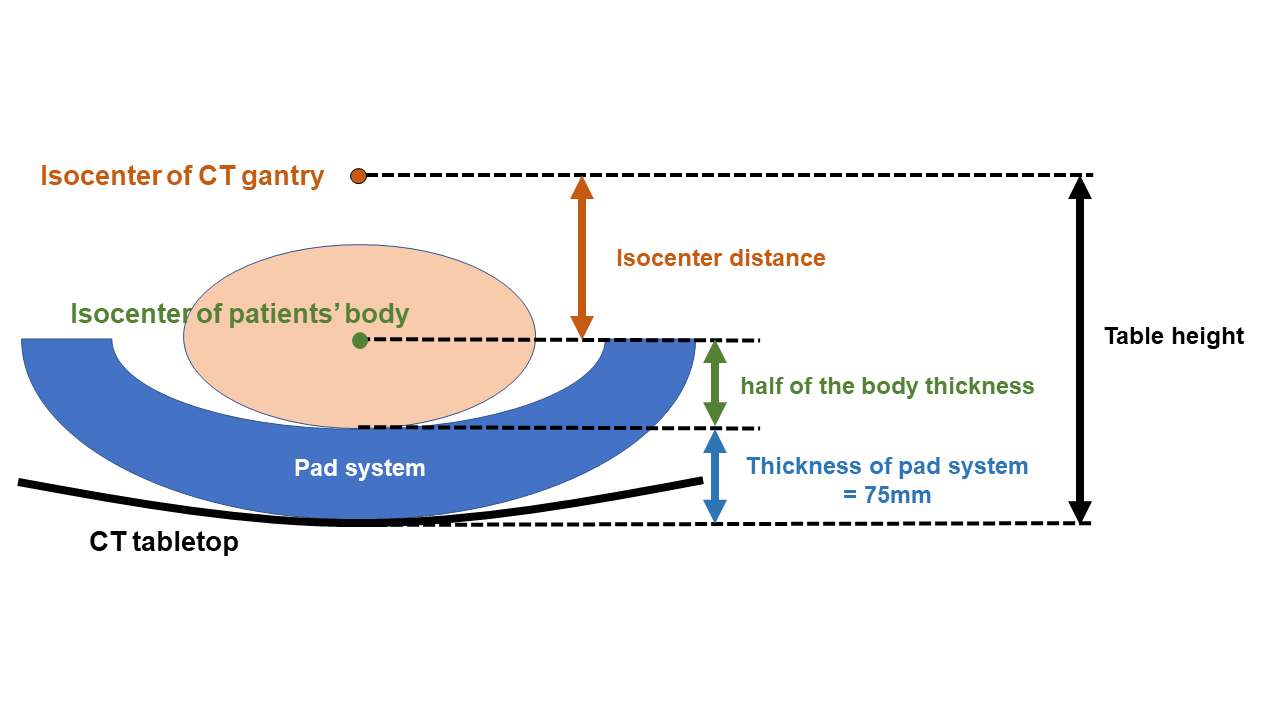

Supplement: Supplementary file 1 — Supplementary Figure S1. [file 41598_2022_21018_MOESM1_ESM.tif]
