# Supplementary material for: Patient positioning during pediatric cardiothoracic computed tomography using a high-resilience pad system and pre-scan measurement of chest thickness
Source: Sci Rep. 2022 Oct 5;12:16618. doi: 10.1038/s41598-022-21018-5 (PMC9534888; doi:10.1038/s41598-022-21018-5)
Supplement: Supplementary file 2 — Supplementary Legends. [file 41598_2022_21018_MOESM2_ESM.docx]

**Supplemental Figure Caption**

**Title: Definition of each distance**

The table height and isocenter distance are defined as the distance from the isocenter of the CT gantry to CT tabletop and to the isocenter of patient’s body, respectively. The pad system was scanned and measured its total thickness as 75 mm before this study.
